# Supplementary material for: Magnitude of consistent condom use and associated factors among people living with HIV/AIDS in Ethiopia: Implication for reducing infections and re-infection. A systematic review and meta-analysis
Source: PLoS One. 2024 Jul 29;19(7):e0304248. doi: 10.1371/journal.pone.0304248 (PMC11285934; doi:10.1371/journal.pone.0304248)
Supplement: S1 File — (DOCX) [file pone.0304248.s002.docx]

**Search strategies**

**PUBMED**

(((Magnitude[All Fields] OR ("condoms"[MeSH Terms] OR "condoms"[All Fields])) AND (Associated[All Fields] AND factors[All Fields])) AND HIV/AIDS[All Fields]) AND ("ethiopia"[MeSH Terms] OR "ethiopia"[All Fields])

**SCOPUS**

Aditional searching(ALL(magnitude)AND ALL(consistent condom) AND (Associated factors) AND TITLE-ABS-KEY(HIV/AIDS) TITLE-ABS-KEY(Ethiopia) were used
