# Supplementary material for: Magnitude of consistent condom use and associated factors among people living with HIV/AIDS in Ethiopia: Implication for reducing infections and re-infection. A systematic review and meta-analysis
Source: PLoS One. 2024 Jul 29;19(7):e0304248. doi: 10.1371/journal.pone.0304248 (PMC11285934; doi:10.1371/journal.pone.0304248)
Supplement: Attachment — Submitted filename: Magnitude of consistent condom use and associated factors among peoples living with.docx [file pone.0304248.s003.docx]

Magnitude of consistent condom use and associated factors among peoples living with

HIV/AIDS in Ethiopia: Implication for reducing infections and re-infection. A systematic

review and meta-analysis

This paper explores extent of consistent condom use and factors associated with consistent condom use among people living with HIV in Ethiopia. Although the topic is important, there are several issues in presentation of the data. There has been inconsistent use of the language, and language appropriateness. Overall, Although methodologically correct, It needs to be thoroughly reviewed by an English speaker. Below are few examples of the areas need to be revised, however, there is a lot more than what I singled out.

Comments

Abstract:

Results section there is a sentence “ the pooled estimate of **self-care** (What is self-care? It has never been defined earlier**.** I expected pooled prevalence of consistent condom use instead of self-care)

Conclusion: This study **shown** that (it should be showed)

“telling their sexual partners about their” Disclosing HIV status is better than telling their partners…..

Background:

…..is dependent on the consistent and proper use of condoms(2, 4-6). **However,** people who started (There is no connection with the use of word **however** here.)

Sub-Saharan Africa, especially Ethiopia, has a very high incidence of the problem (2). (Better state it explicitly instead of referring it as a problem

Despite **a** variable reports of magnitude and…… (Grammatically incorrect)

Methods

The objective of the review was to conclude the magnitude and associated factors of consistent

condom use among peoples living with HIV/AIDS in Ethiopia (This needs revision such as ……..magnitude of consistent condom use and factors associated with consistent condom use……..)

MESH term for the database **is** ( should be MESH term for the database **were…..)**

**Discussion**

The rate of adherence ….. (Adherence to what? better use same word consistent cdm use.)

patients who reported their status (disclosed? reported to who?)

This result was at odds with the research on Cameroon (I don’t understand what is meant here)
