# Supplementary material for: Magnitude of consistent condom use and associated factors among people living with HIV/AIDS in Ethiopia: Implication for reducing infections and re-infection. A systematic review and meta-analysis
Source: PLoS One. 2024 Jul 29;19(7):e0304248. doi: 10.1371/journal.pone.0304248 (PMC11285934; doi:10.1371/journal.pone.0304248)
Supplement: Attachment — Submitted filename: Condom RESPONSE FINAL.docx [file pone.0304248.s004.docx]

Chuanyi Ning, Ph.D.

Academic Editor of PLOS ONE

Dear Editor of the Manuscript PONE-D-23-16392 Magnitude of consistent condom use and associated factors among peoples living with HIV/AIDS in Ethiopia: Implication for reducing infections and re-infection. A systematic review and meta-analysis" submitted to PLOS ONE. Thanks for your time and consideration in editing and reviewing the manuscript. We have carefully read your comments and corrected inline of your comments and suggestions. All comments raised were edited and incorporated in the revised manuscript.

Here are the responses and elaborations for the comments from the editor and reviewer!

**REVIEWERS COMMENT**

**Reviewer 1:**

**Reviewer comment:** several distinct systematic reviews and meta-analyses regarding risky sexual behaviour among HIV/AIDS patients have been conducted in Ethiopia. These studies used a similar operational definition and cited inconsistent condom use as a risky sexual practice. Additionally, I was unable to gain any new information or insights from this study, and the authors were unable to present concrete evidence that there was a literature gap or any other factors that made the current research different from previously published publications.

Author response: Risky sexual behaviour is the general term but inconsistent condom use is too specific term. Therefore, those two studies are quite different that makes our study unique from risky sexual behaviour. Many authors defined risky sexual behaviour as having multiple sexual partners, early initiation of sex, inconsistent condom use, sex with commercial sex workers, and unprotected sex with a same-sex partner, especially when it involves male partners

**Reviewer comment:** The authors used the terms "adherence to self-care" and "consistently used condoms" interchangeably; I'm not sure these terms are similar, and I believe they have different meanings in this context. Consistent condom use may be one self-care practise that people with HIV/AIDS engage in, and self-care is a holistic approach. I advise the authors to employ one of them consistently throughout the whole paper.

Author response: We have used consistent condom use instead of adherence to self-care throughout the paper

**Reviewer comment:** What are the gaps? I've noticed that the introduction is very poor and insufficient, and there is no clear, updated context about the current situation of HIV/AIDS in Ethiopia. Moreover, the report fails to explain how it differs from earlier comparable systematic reviews and meta-analyses. Additionally, most of the references are incorrectly cited. For instance, the authors used reference number 3 to illustrate the prevalence of HIV/AIDS in Ethiopia, but the cited study was unable to provide this data; as a result, the correct data for this information can be found in this article/Ababa A. Ethiopia. Abstract available from: [https://wfpha.confex.com/wfpha/2012/webprogram/Paper10587.html. 2013](https://wfpha.confex.com/wfpha/2012/webprogram/Paper10587.html.%202013).

Author response: The revised manuscript contains of the study gaps, and sufficient background information. The updated data on the current situation of HIV/AIDS was added.The difference between the pervious study and our study was stated in introduction part as ”In Ethiopia, there was no systematic review and meta-analysis was conducted regarding the magnitude and predictors of consistent condom use, and the previous comparable systematic reviews and meta-analyses done was on risky sexual behavior that includes having multiple sexual partners, early initiation of sex, sex with commercial sex workers, and unprotected sex with a same-sex partner, which is different from consistent condom use. Our study only reports on condom utilization patterns. Therefore, the purpose of this paper is to summarize the recent findings on magnitude and factors related to consistent condom use in order to provide an appropriate intervention”.

The reference no 3 was replaced by another updated references

**Reviewer comment:**  The author said that the review procedure was registered on PROSPERO CRD430396 in the method section. However, the registration digits were missing; therefore, if the review was registered, I advise the author to supply the whole registration number.

Author response: The review was registered and given a registration no of PROSPERO CRD42023430396

**Reviewer comment:**  Search strategies: It would be good practice to include the search strategies and the number of hits for each line as an appendix or supplementary material.

Author response: The Search strategies was supplied as supplementary material in revised manuscript

**Reviewer comment:**  If possible, could you elaborate on the inclusion and exclusion criteria under the heading "Eligibility criteria" for the review? How about the primary study's choice of study design?

Author response: We have clearly explain it in revised manuscript

**Reviewer comment:**  The Joanna Briggs Institute Meta-analysis of Statistics Assessment and Review Instrument (JBIMAStARI) was used by the authors to evaluate the quality of their work; however, they were unable to describe how to utilise it. no explanation of the criteria used for assessing quality. When do you say good quality article?" Could you please describe how to use the tool? How many reviewers, for instance, completed the quality assessment? Additionally, I didn't see any reports about quality evaluation in the results section.

Author response: We have corrected in quality assessment section of revised manuscript as” The criteria used for assessing the quality of included studies were as follows; Were the criteria for inclusion in the sample clearly defined?, Were the study subjects and the setting described in detail?, Was the exposure measured in a valid and reliable way?, Were objective, standard criteria used for measurement of the condition?, Were confounding factors identified?, Were strategies to deal with confounding factors stated?, Were the outcomes measured in a valid and reliable way?, and Was appropriate statistical analysis used?. The quality assessment were done by two authors (FB and GB) using a quality assessment checklist. Discrepancies between two independent reviewers were resolved by involving third reviewers (TD) after discussion for possible consensus. Accordingly, the articles was classified as high quality if the score is >80%, moderate if (65-80%), and low if <65%. The reports about quality evaluation was described in table 2 of revised manuscript under results section.

**Reviewer comment:**  In this final method part subheading, authors wrote about the risk of bias. I'm not sure what the authors are saying, it sounds like personal opinion. Please provide concise support and write it better in the analysis portion with the proper reference.

Author response: The risk of bias section is omitted and stated as limitation in revised manuscript

**Reviewer comment:**  The data analysis and synthesis sections were extremely short, and all the plans utilised were not cited. In addition, the authors didn't specify which model to apply (fixed effect or random); what about the pan-sub-group and sensitivity analyses? What is the evidence for claiming heterogeneity at a p-value of 0.05?

Author response: The data analysis section was revised to include all the plan and model used, sub-group analysis and sensitivity analysis

**Reviewer comment:**  in the result, according to the criteria in the method section, your study's heterogeneity was moderate. What does it stand for? Describe it in detail, please. Describe the probable effects of this moderate degree of heterogeneity on the pooled prevalence estimate and discuss any possible causes of this moderate level of heterogeneity.

Author response: We have corrected in revised manuscript as “The moderate heterogeneity showed the presence of moderate variations across studies rather than by chance. This heterogeneity can alter the pooled magnitude estimate of consistent condom use. Therefore, subgroup analysis and sensitivity analysis was carried out. The region was the possible causes of this moderate heterogeneity. The possible reasons for heterogeneity, other than clinical differences, might include methodological issues such as problems with sampling technique”.

**Reviewer comment:**  In discussion section, how did the authors ensure that, to the best of their knowledge, this meta-analysis and systematic review are the first of their kind to be conducted at the national level in Ethiopia to estimate the magnitude and identify the factors associated with consistent condom use among HIV/AIDS patients, despite the fact that many other studies of a similar nature have been carried out in Ethiopia?

Author response: There was no systematic review and meta-analysis study conducted on magnitude and associated factors with consistent condom use among HIV/AIDS patients in Ethiopia. The already conducted study was on risky sexual behavior which is different from our study

**Reviewer comment:**  The discussion portion is too short and lacks comparison of the details with the existing evidence. The authors failed to highlight the clinical implications, and I was unable to locate the implications of this study for stakeholders and policymakers. They also made no recommendations for these groups of people.

Author response: The discussion was modified and extensive information was added. Besides, the clinical implications and recommendation were added.

**Reviewer comment:**  Finally, I advise the author to thoroughly review every area of the work and seek the advice of professionals in grammar and language.

Author response: The whole document was revised and the grammar was edited by one fluent English speaker.

**Reviewer 2:**

**Reviewer comment:**  There has been inconsistent use of the language, and language appropriateness. Overall, Although methodologically correct, It needs to be thoroughly reviewed by an English speaker.

Author response: The whole document was revised and the grammar was edited by one fluent English speaker.

**Reviewer comment:**  Results section there is a sentence “ the pooled estimate of self-care (What is self-care? It has never been defined earlier. I expected pooled prevalence of consistent condom use instead of self-care)

Author response: The word self-care was replaced by consistent condom use

**Reviewer comment:**  Conclusion: This study shown that (it should be showed)

Author response: It was corrected as showed

**Reviewer comment:**   “telling their sexual partners about their” Disclosing HIV status is better than telling their partners…..

Author response: We have corrected as Disclosing HIV status.

**Reviewer comment:**  .is dependent on the consistent and proper use of condoms(2, 4-6). However, people who started (There is no connection with the use of word however here.)

Author response: The sentence was re-written in revised manuscript

**Reviewer comment:**  Sub-Saharan Africa, especially Ethiopia, has a very high incidence of the problem (2). (Better state it explicitly instead of referring it as a problem

Author response: We have mentioned the problem in the revised manuscript

**Reviewer comment:**  Despite a variable reports of magnitude and…… (Grammatically incorrect)

Author response: We have corrected its grammar in the revised manuscript.

**Reviewer comment:** The objective of the review was to conclude the magnitude and associated factors of consistent
condom use among peoples living with HIV/AIDS in Ethiopia (This needs revision such as ……..magnitude of consistent condom use and factors associated with consistent condom use……..)

Author response: We have revised it as per your comment

**Reviewer comment:** MESH term for the database is ( should be MESH term for the database were…..)

Author response: Is was replaced by were

**Reviewer comment:** The rate of adherence ….. (Adherence to what? better use same word consistent cdm use.)

Author response: We have replaced adherence by consistent condom use

**Reviewer comment:** patients who reported their status (disclosed? reported to who?)

Author response: We have corrected as patients who disclosed their status to their partner in revised manuscript

**Reviewer comment:** This result was at odds with the research on Cameroon (I don’t understand what is meant here)

Author response: We mean different. Therefore, we have replaced odds by different in revised manuscript

Thanks for your time and consideration,

Regards!
