# Supplementary material for: Magnitude of consistent condom use and associated factors among people living with HIV/AIDS in Ethiopia: Implication for reducing infections and re-infection. A systematic review and meta-analysis
Source: PLoS One. 2024 Jul 29;19(7):e0304248. doi: 10.1371/journal.pone.0304248 (PMC11285934; doi:10.1371/journal.pone.0304248)
Supplement: Attachment — Submitted filename: Comments_11NOV2024.docx [file pone.0304248.s005.docx]

Overall, there is some improvement however there are still several grammatical errors. thoroughly review by an English speaker is recommended. Below are few examples of the areas need to be revised, however, there is a lot more than what I singled out.

**Revised version reviewed.**

**Title; Change peoples to people**

Abstract: More than one million peoples…. (change peoples to people)

….among HIV patients…., change it to people living with HIV or people with HIV

We have used four databases such as PubMed, when you say such as is like you are giving examples of the databases but not exact databases you searched information. Revise this sentence because those databases are the one you sought information

Main text

Methods: …Accordingly, the articles was (should be article was or articles were)

Results …majority of the participant was female in all articles ( should be majority of participants were..)

….who were disclosed their status was 5.61 time … (should be who disclosed their status…)

…patients who were live in urban area was 3.46 time (should be … who live in urban area were 3.46 times … or who are living in urban area were …..)

…patients who were married was 67% times ( should be patients who were married were 67% times…)

In addition, the measure of effect used was odd ratio, however, the interpretation is like risk ratio.

It should be something like people living in urban areas have higher odds or reporting consistent condom use compared to rural areas. Not saying people living in urban areas are more likely reporting consistent condom use compared to rural areas.
