# Supplementary material for: Magnitude of consistent condom use and associated factors among people living with HIV/AIDS in Ethiopia: Implication for reducing infections and re-infection. A systematic review and meta-analysis
Source: PLoS One. 2024 Jul 29;19(7):e0304248. doi: 10.1371/journal.pone.0304248 (PMC11285934; doi:10.1371/journal.pone.0304248)
Supplement: Attachment — Submitted filename: Condom use PLOS ONE RESPONSE FINAL.docx [file pone.0304248.s006.docx]

AUTHOR RESPONSE LETTER

**Nagasa Dida, MPH**

Academic Editor of PLOS ONE

Dear Editor of the Manuscript PONE-D-23-16392R1 entitled “Magnitude of consistent condom use and associated factors among peoples living with HIV/AIDS in Ethiopia: Implication for reducing infections and re-infection. A systematic review and meta-analysis" submitted to PLOS ONE. Thanks for your time and consideration in editing and reviewing the manuscript. We have carefully read your comments and corrected inline of your comments and suggestions. All comments raised were edited and incorporated in the revised manuscript.

Here are the responses and elaborations for the comments from the editor and reviewer!

**REVIEWERS COMMENT**

**Reviewer 1:**

**Reviewer comment:** The authors noted that there was conflicting evidence available in Ethiopia about the prevalence and correlates of frequent condom usage in the abstract portion of the paper. Keeping this in mind, how do the authors address these problems? How do they generate fresh evidence? What were the conflicting issues that appeared in earlier literature?

Author response: There was no conflicts of ideas but we mean to explain in revised manuscript as there is inconsistent reports of the prevalence and correlates of frequent condom use in Ethiopia.

**Reviewer comment:** The abstract's method section has to be updated in light of the objectives: How was consistent condom usage measured and reported? How were the factors associated with condom use described and their statistical significance determined? How about sub-group analysis carried out by region or any other criteria, too? How was the objective publication bias determined?
Author response: We have updated abstract's method section in line with our objectives in revised manuscript

**Reviewer comment:** The numbers on the number of people living with HIV/AIDS needed to be reported along with the year, as stated in the first and second paragraphs of the introduction. At what time did this report occur?
Author response: The year of report was added

**Reviewer comment:** - In the introduction section, the authors noted that different countries use condoms at varying rates. To get a better general picture of condom usage among HIV/AIDS patients, I asked the authors to describe the prevalence of condom use among people living with the disease elsewhere in the world, with a focus on sub-Saharan Africa and Ethiopia.
Author response: We have added many literature across world with focusing on sub-Saharan Africa in revised manuscript

**Reviewer comment:** - be more appropriate to cite the prior comparable reviews and analyses that have concentrated on risky sexual activity, which is different from your work on regular condom use? Could you offer any alternative suggestions to the ones found in the earlier studies? On the other hand, tackling risky sexual behavior entails encouraging frequent condom usage, right? Could there be different results? I believe that all of your research and earlier studies focused on preventing HIV/AIDS and re-infection.
Author response: We have compared our study with many previous reviews in revised manuscript. There was a difference between risky sexual behavior and consistent condom use according to our study.

**Reviewer comment:** - on objectives: The objective of the review was to conclude the magnitude and associated factors of consistent condom use among people living with HIV/AIDS in Ethiopia. It is better to say to detrmine instead of to conclude.
Author response: We have correct it as per your comments

**Reviewer comment:** - on search strategies: It would be best practice to include the search methods and the quantity of results for each database as an appendix or supplemental material. However, the authors either didn't do that or we don't have access to the supplemental table outlining the search methods and findings for each database.
Author response: We have included the search methods as supplemental material in previous revision

**Reviewer comment:** - Why a publication year-based subgroup analysis? Does it affect the study in any way?

Author response: The study only differ by publication year and region. The publication year can affects the outcome

**Reviewer comment:** - The discussion part still requires improvement and a more thorough comparison with past similar reviews and meta-analyses conducted worldwide. The comparison between this pooled prevalence and other studies' point prevalence was the authors' sole focus. When comparing the pooled data with point prevalence, is it good?
Author response: We have revised our discussion and it was compared with the reviews and meta-analyses conducted worldwide. We have compared the pooled data with the pooled data in majority of the cases. However, a few comparison was used for point prevalence in the case of shortage of literatures.

**Reviewer comment:** - On the other hand, the authors calculated the pooled odds ratio of factors affecting consistently using condoms in Ethiopia. then continue comparing the findings to those of another study done in Ethiopia (Dessie)? What caused it? What's the point? They neglected to include this study in their evaluation.
Author response: We have mistakenly compared with the study done in Ethiopia (Dessie).We have included the study in our analysis.

**Reviewer comment:** - In relation to the study's implications. Pharaphrasing and citations are necessary.

Author response: We have cited and paraphrased it

**Reviewer comment:** - The authors needed to describe the sub-group analysis by region in the discussion section for better clarity, policy implications, and to identify the region of requirements (regional variance). can explain the differences as well as make recommendations for areas with low prevalence?

Author response: We have discussed the difference in the consistent condom use across a different regions. We also put a recommendation for lower prevalence.

**Reviewer comment:**- In the limitation section? Does your study represent the whole region of Ethiopia? Are all regions of Ethiopia represented?

Author response: We have put as limitation that all regions of Ethiopia were not included

**Reviewer comment:**- The conclusion is far too short. Try to address all of the key findings and make workable suggestions.

Author response: We have added some important findings in conclusion part

Reviewer 2:

**Reviewer comment:** Title; Change peoples to people

Author response: We have changed it

**Reviewer comment:** Abstract: More than one million peoples…. (change peoples to people)
….among HIV patients…., change it to people living with HIV or people with HIV
We have used four databases such as PubMed, when you say such as is like you are giving examples of the databases but not exact databases you searched information. Revise this sentence because those databases are the one you sought information
Author response: We have changed the peoples to people, among HIV patients is change to people living with HIV, the word such as is like is replaced by word explaining the exact data base

**Reviewer comment:** Main text
Methods: …Accordingly, the articles was (should be article was or articles were)
Results …majority of the participant was female in all articles ( should be majority of participants were..)
….who were disclosed their status was 5.61 time … (should be who disclosed their status…)
…patients who were live in urban area was 3.46 time (should be … who live in urban area were 3.46 times … or who are living in urban area were …..)
…patients who were married was 67% times ( should be patients who were married were 67% times…)

Author response: We have incorporated all your comments in revised version

**Reviewer comment:** In addition, the measure of effect used was odd ratio, however, the interpretation is like risk ratio.
It should be something like people living in urban areas have higher odds or reporting consistent condom use compared to rural areas. Not saying people living in urban areas are more likely reporting consistent condom use compared to rural areas.

Author response: We have described the associated factors by odds ratio in revised manuscript

**Reviewer 3:**

**Reviewer comment:** Thank you for addressing the points highlighted in the previous review. The manuscript reads well and is technically sound.

Author response: Thank you for your comment

Thanks for your time and consideration,

Regards!
